# Development of a novel social incubator for health promoting initiatives in a disadvantaged region

**DOI:** 10.1186/s12889-020-08990-1

**Published:** 2020-06-10

**Authors:** Sivan Spitzer-Shohat, Jumanah Essa-Hadad, Mary CJ Rudolf

**Affiliations:** Department of Population Health, Azrieli Faculty of Medicine, Bar-Ilan University, POB 1589, Henrietta Szold 8, 1311502 Safed, Israel

**Keywords:** Health inequity, Social incubator, Community based participatory research

## Abstract

**Background:**

Bottom-up approaches to disparity reduction present a departure from traditional service models where health services are traditionally delivered top-down. Raphael, a novel bottom-up social incubator, was developed in a disadvantaged region with the aim of ‘hatching’ innovative health improvement interventions through academia-community partnership.

**Methods:**

Community organizations were invited to submit proposals for incubation. Selection was made using the criteria of innovation, population neediness and potential for health impact and sustainability. Raphael partnered with organizations to pilot and evaluate their intervention with $5000 seed-funding. The evaluation was guided by the conceptual framework of technological incubators. Outcomes and sustainability were ascertained through qualitative and quantitative analysis of records and interviews at 12 months and 3–5 years, and the Community Impact of Research Oriented Partnerships (CIROP) questionnaire was administered to community partners.

**Results:**

Ninety proposals were submitted between 2013 and 2015 principally from non-governmental organizations (NGOs). Thirteen interventions were selected for ‘incubation’. Twelve successfully ‘hatched’: three demonstrated sustainability with extension locally or nationally through acquiring external competitive funding; six continued to have influence within their organizations; three failed to continue beyond the pilot. Benefits to the organisations included acquisition of skills including advocacy, teaching and health promotion, evaluation skills and ability to utilize acquired knowledge for implementation. CIROP demonstrated that individuals’ research skills were reported to improve (mean ± sd) 4.80 ± 2.49 along with confidence in being able to use knowledge acquired in everyday practice (5.50 ± 1.38) and new connections were facilitated (5.33 ± 2.25).

**Conclusions:**

Raphael, devised as a ‘social incubator’, succeeded in nurturing novel ideas engendered by community organizations that aimed to impact on health disparities. Judging by success rates of technological incubators its goals were realized to a considerable degree.

## Background

Health disparities are pervasive and can be identified at all stages of the life cycle, from birth to old age [1]. The widening gaps in both access and provision of care have triggered a search for effective strategies for reducing disparities, generally characterized as either top-to-bottom or bottom-up [2, 3]. The top-down approach to disparity reduction focuses on health system redesign and the way in which providers can improve health outcomes by improving organisational performance [4, 5].

Bottom-up approaches aimed at disparity reduction focus mainly on the tailoring of care processes to the needs of individual patients or specific communities [2, 6]. These strategies represent a departure from the traditional service model viewing the community simply as recipients of health services, to community engagement in which the community is an active partner in improving health [7–9].

Over the last two decades, one of the most common strategies of community engagement utilized for reducing health disparities is Community Based Participatory Research [10–12]. Community-based participatory research (CBPR) aims at creating transformative research through partnering between academia and the community [13, 14]. In CBPR there is a commitment to feeding back, interpreting, disseminating, and translating the data jointly to create interventions and/or policy which can better the health of the community [15].

Studies report that the formation of such partnerships have been challenging due to the lack of skills and experience of community partners in conducting proper research [16]. Moreover, CBPR partnerships are complex and often encounter barriers such as lack of trust among partners, difficulty in translating from the lab into ‘real world’, difficulty in changing traditional perceptions on the one-directional flow of knowledge from academia to the community, and difficulty in achieving sustainability [10].

The current paper describes the implementation of Project RAPHAEL, a novel form of CBPR, which was designed as a ‘social incubator‘ for improving health in Israel’s disadvantaged northern periphery. The Raphael model draws from the framework of technological incubators and aims to create a supportive environment that is conducive to the “hatching” and development of new interventions to reduce health disparities [17–19]. Over the years, technological incubators have become a ubiquitous phenomenon, creating a platform for academia and business, to promote economic development, innovativeness and the emergence of new technology-based growth firms [20]. Through the combination of practices and professional norms, incubators have been found to promote ‘value added ties’ in which universities mentor projects, offer access to research facilities as well as gain knowledge on what is happening in the field, while companies gain knowledge skills and scientific credibility [21].

The aim of this paper is to describe the RAPHAEL social incubator model and the extent to which it succeeded not only in fostering and nurturing community-based ideas, but in training and skilling up local organisations so that the intervention formulated had the potential to be sustainable over time.

## Methods

### Setting

The Azrieli Faculty of Medicine was established 7 years ago in the city of Safed in the Galilee, which has among the poorest markers for health and least accessibility to health care in Israel [22, 23]. Over 37% of the population lives in poverty, and the majority of towns in the region rate lower than 5 on the national socio-economic 10 step ladder [24]. A significant proportion of the country’s most disadvantaged populations live in the Galilee, and are characterized by low income families, low education levels, and high unemployment. The population is diverse comprising communities of ultraorthodox Jews, Ethiopian and Russian immigrants, Druze, and Christian and Muslim Arabs.

### Conceptual framework

Our evaluation was guided by the conceptual framework of technological incubators including: (1) Selection of projects; (2) Support including coaching/training activities undertaken to develop the incubatees, (3) Mediation which refers to the intra-relationships between different projects within the incubators as well as the inter-relationships to the outside world, and (4) Graduation/Hatching which assesses what happens to the projects upon finishing the incubation processes [20].

#### Phase 1: mapping of submissions

The first phase of the project involved a call for proposals. This had a dual purpose: to draw in projects for piloting the incubator, and to map health needs from the community perspective. In 2013, 2014 and 2015 a call was disseminated by email, social media, personal contacts and word of mouth to municipalities, NGOs, health maintenance organisations, universities, hospitals, schools, and other community organisations in the region. They were asked to describe the needs of their population and were invited to submit their ideas for a project that they believed would improve health. The call emphasized that the term health was not limited to medical problems and health services.

The submissions received were analysed by the Raphael team considering their focus, the characteristics of the population targeted by the respondents, the location of the proposed project and the type of organisation submitting the idea.

#### Phase 2: selection process

Applications were independently reviewed by the Raphael team and scored according to the following criteria: creativity and innovation; neediness of the target population; potential impact on health or welfare; potential for sustainability; and potential for replication elsewhere. Proposals of interest were discussed with the Raphael community advisory group, which included stakeholders from the local municipality and regional councils, regional Ministry of Health, and local NGOs. In each cycle 10 to 12 organisations were invited for an in depth interview of which 4 or 5 were selected to participate in the incubator.

#### Phase 3: mediation - partnership working

Each of the selected organisations was paired with a coordinator (SS or JE) from the Raphael team whose task was to provide facilitation and academic expertise. This involved helping to ascertain the health needs of the community, finding research evidence to inform the proposed intervention, developing an evaluation framework and accompanying the project during its pilot.

The organisations were brought together every 4 months to engage in dialogue, share experiences, and learn from the know-how and practical experience of each other. They were also provided with $5000 in seed funding. On completion of the projects, meetings were held with each organisation to discuss the outcome of the project and help plan for ongoing sustainability.

#### Phase 4: mixed methodology evaluation

Organisation representatives completed the Community Impact of Research Oriented Partnerships (CIROP) questionnaire at the end of the incubation period. CIROP is a 33-item tool measuring the extent the partnership improved knowledge and research skill development, organisational development and access/use of information (on a Likert scale of 1–7) [25]. The questionnaire includes 3 open ended questions on major areas of impact; areas of least impact and general comments. Quantitative data was analysed descriptively, and the open-ended questions were analysed for themes.

At the end of the four-year period an overview was carried out by the team assessing the mediation and graduation/hatching of the projects. We assessed the success of the pilot, sustainability in the organisation beyond the end of the project, extension beyond the local organisation, the quality of partnership working and any benefits the coordinator perceived for the organisations. The information was attained from the academic coordinator’s detailed notes as well as from key informants, with the organisations where necessary, and underwent an explanatory content analysis.

Telephone interviews were also conducted in January 2019 to ascertain the extent to which projects had been sustained and whether they were ongoing in any form within or beyond the organisation. A member check [26] was also conducted to receive their feedback on the Raphael overview.

Finally, three researchers reviewed the projects independently using a 5-point scale to score the projects’ implementation: 1 - extended beyond the base site / 2- sustained locally / 3 – incompletely sustained but some ongoing influence / 4 - not sustained / 5 - failure to implement. The ratings were discussed until interrater reliability was attained.

## Results

### Mapping of submissions

A total of 90 applications were received between 2013 and 2015. Table 1 shows the focus of the proposals, their target populations, the type of organisation and proximity to the medical school. Organisations responding to the call were for the most part NGOs from across the Region, although local municipalities also featured; there was surprisingly little response from the health sector. Almost half the proposals focused on lifestyle and improving well-being. The remainder related to long term medical conditions and preventive medicine with a minority addressing health services and the environment.

**Table 1:** Analysis of 90 proposals submitted to the social incubator

	Submitted proposals *n* = 90	Selected proposals *n* = 13
**Primary focus of the project**		
Lifestyle	21 (23%)	1
Well-being/mental health	16 (18%)	3
Mental illness	12 (13%)	4
Disability	11 (12%)	2
Preventive medicine (screening, child injury prevention, infectious diseases)	9 (10%)	2
Chronic illness (including cancer)	6 (7%)	–
Sexual abuse and violence	6 (7%)	–
Health services quality improvement	6 (7%)	–
Environmental issues	3 (3%)	1
**Ethnicity of target population**		
Jewish	33 (37%)	5
Arab	25 (28%)	3
Haredi	3 (3%)	1
Mixed Jewish Arab	29 (32%)	4
**Submitting organisation**^a^		
NGO	56 (59%)	6
Local municipality	14 (15%)	5
Hospital	11 (11%)	1
University	6 (6%)	–
Private initiatives	5 (5%)	–
HMO	2 (2%)	–
School	2 (2%)	1
**Proximity to the medical school**^a^		
Local to Safed	23 (24%)	8
Within 30 km radius of Safed	6 (6%)	1
More than 30 km radius of Safed	67 (70%)	5

^a^Total > 90 as some projects were submitted by 2 collaborating organisations

### Selection for participation in the social incubator

Over the course of 3 cycles, 13 proposals were selected to participate in Raphael (see Table 1). Over half related to mental health or illness, and targeted both Jewish and Arab populations. The community advisory board proved to be invaluable, particularly regarding the local knowledge they provided about the submitting organisations. Table 2 provides details of the selected partners, the projects and the innovative aspects that influenced their selection.

**Table 2:** The Raphael Social Incubator: Participants, projects and their outcome

Partner	Project	Innovative aspects	Outcomes	Roll Out Beyond the Local Organisation	External funding received to extend the project	Deemed as a Social Incubator Success
**Social incubator success (rating 1)**						
Local HMOs with Hatzor Social Welfare Services	Shared Mental Health Care	Creating an inter-agency shared care model across medical and social services for socially disadvantaged patients with mental illness	Balint groups established. 240 individuals received care through the project with reduction in health services use and medications	Three additional clinics established in southern and northern peripheries. Working with 3 of Israel’s 4 HMOs	Prestigious research grant from the Israeli National Institute of Health Policy Research	Yes - sustained and extended nationally
Al-Manal, an NGO supporting Arab women, with Dugrinet, an NGO providing media training for charities	Empowering disabled Arab women as agents for health	Drawing on the potential strengths of isolated physically disabled Arab women to advocate for enhanced health services required by the disabled	Well-established team of 10 disabled Arab women. Audiovisual materials produced and meetings held with health service managers and in the community. Enhanced social circumstances for participants and self-esteem enhanced.	Another national NGO for physically disabled Arabs adopted parts of the program.	Grant from USA embassy MEPI program Funding from the NGO Healing across the Divides	Yes - sustained and extended locally
Beterem- Safe Kids Israel (NGO promoting child safety)	Community Champions for Child Safety	Creating a team of volunteers in disadvantaged Arab and Jewish communities to ‘champion’ child safety on postnatal wards and in kindergartens	Safety lectures to new mothers directly after birth delivered weekly at Nazareth hospital. Volunteers also visit local schools to give session on safety to students and staff.	Extended to another neighbouring Arab towns	Funding from the NGO Healing across the Divides, and from Generation to Generation	Yes - sustained and extended locally
**Successfully ‘hatched’ with influence ongoing in some form (rating 2–3)**						
Enosh, an NGO for individuals coping with mental illness	Lifestyle program for individuals coping with mental illness	Hiking as a regular and core activity to engender lifestyle change in the face of severe mental illness	20 active participants on hikes. Healthy changes also engendered in club environment: healthier foods, more physical activity. Medical students are now involved.	The initiative influenced the adoption of healthy lifestyle in Enosh branches nationally	Local lifestyle program is now supported by National Enosh and private donors	Continued within Enosh locally
Safed Municipality- social welfare services	Clowning by the elderly for the elderly	Creating a team of elderly clowns and bringing medical clowning to the community setting	A dedicated team of clowns trained, reporting increased meaning in their own lives. Visits made to local retirement homes and other community organisations are ongoing to an extent	_	Prize received	Ongoing influence in Safed social services
Carmiel Municipality Employment Office	Factory “health champions”	Establishing a network of trained factory workers to be ‘health champions’ within their factories	Four factories engaged. Changes in all four reported: eg healthier food options available in cafeterias; smoking policies and designated smoking areas outside factory instated.	_	_	Some ongoing influence in the factories
Al-Taj Arab High School (NGO), Arrabeh	Arab high school girls as agents for health change	Training high school Arab girls to work inter-generationally to promote breast cancer awareness and mammography uptake	44 students trained and reported positive conversations with women in their families. Ongoing lectures within school but no direct work with families	_	_	Some ongoing influence in the school
Menachem Begin Amal High School in a low SES area	Enhancing self-esteem in high school students	Combining the drama curriculum with school psychology support services and community service in order to promote self esteem	Program well received with drama and counseling workshops on-going beyond the pilot year. No measurable increase in self-esteem found.	_	_	Some ongoing influence in the school.
Child Development Center- Ziv Hospital	Autism: Play Project	Culturally adapting a USA evidenced based program for Arab and Jewish families	22 families enrolled. Therapists gained skills. Marked improvement in play skills and parent-child communication in 8 families completing the course. Approach still used in Center but no home visits	_	_	Some ongoing influence in the Child Development Center
**Successfully hatched but not sustained (Rating 4)**						
ENOSH, an NGO for individuals coping with mental illness	“Phototherapy” in mental illness	Increasing community awareness of mental illness and encouraging self-expression of individuals coping with severe mental illness through teaching photography in the framework of an NGO	7 members completed the photography course and exhibited their work to the public in Safed City Arts Center.	_	_	Successful implementation but not sustained
ENOSH Safed, NGO for individuals with mental illness.	Using Jewish sources to engage ultraorthodox men coping with mental illness	Engaging ultraorthodox Jews to cope with their severe mental illness in a culturally specific way, in a community where mental illness is often hidden	13 Haredi men with mental illness attended a 12 week course with positive benefits.	_	_	Successful implementation but not sustained
Upper Galilee Regional Council	Eco-friendly Kibbutz	Developing eco-friendly strategies in garden and play areas on Kibbutzim through raising awareness in the communities’ gardeners	Sixteen gardeners from 5 kibbutzim trained and a strategy implemented for working with the community and farmers to reduce environmental health risks	_	_	Successful implementation but not sustained
**Never ‘hatched’ (Rating 5)**						
Safed Municipality Social Welfare Services	Support group for isolated young Arab mothers	Connecting young mothers from a remote Arab neighbourhood to facilities and activities in the Jewish town	Coordinator appointed but mothers could not be recruited			Failure

### Partnership working

Good partnership working was achieved with all 13 partners, although geographic distance from the Raphael team proved a challenge. The Raphael team primarily took a supportive role, with development of the evaluation process being a significant contribution in all cases. All but one of the projects completed their proposed project, although for most the duration extended beyond the ‘incubation period’ of 12 months.

A total of seven network meetings were implemented at the Faculty over the course of the first 2 cycles. The meetings involved bringing partners together to share their activities with the others, as well as capacity building sessions on organisational development, using communication for disseminating the project, resource development, and project evaluation. These were well attended by local organisations but less so by organisations located outside of Safed. In the third cycle it was evident that partners were struggling to attend due to their geographical distance from the Faculty and so the meetings were discontinued.

### CIROP results

The CIROP questionnaire revealed that although personal research skills only somewhat improved (x = 4.80 sd = 2.49), organisations’ felt that the program increased their confidence in being able to use the knowledge acquired in everyday practice (x = 5.50 sd = 1.38), and facilitated in formation of new connections (x = 5.33, sd = 2.25).

### Qualitative analysis of the overview

The call for proposals achieved its end, with the quantity and richness of the proposals providing a form of mapping of the broad range of concerns and work done by local NGO’s, focused on the needy across all age ranges from both Arab and Jewish communities. The selection process was challenging, and the community advisory board engendered community liaison, which in itself was positive for a young medical school.

All but one project was implemented. All 12 implemented projects were successful in the short term at least, and nine went on to maintain aspects of the project; three of the projects can be considered truly ‘hatched’; they have received grants from external competitive funding sources and have extended their projects locally and even nationally. Details of the projects, their outcomes and success in the long term (along with their scores on the 5-point scale) are provided in Table 2.

Qualitative analysis revealed a variance in priorities with organisations focusing on practicality and implementation with less emphasis on rigorous evaluation. In the words of one manager: “NGOs usually work with funding bodies that demand reports, not research. Research sometimes is the need of the academic partner and does not answer to the needs of the project directly.” (Manager, Org. 1).

Indeed, the focus of the projects proved to be less on research than anticipated, although the evaluation process was central in a number of ways. It generated a greater focus by the community partners on what they were hoping to achieve, it assisted in giving credibility to the project and the community partner and allowed both partners to appreciate the success of the outcome.

A significant success of the partnership was the impact that participation in Raphael had on training of partners’ staff, members and volunteers with skills such as advocacy, teaching, therapeutic approaches or health promotion. It was assumed that partnership working with an academic team would also engender capacity building in the organisations, however, there was little evidence that staff were using more generic skills to develop their organisations. The four monthly meetings of the group were initially a success but were discontinued in the third cycle as only local organisations managed to attend. It was very evident that in almost every case a longer time-frame and more resources were required to extend projects beyond their immediate local organisation.

## Discussion

This paper describes an attempt by a medical school to adapt the technological incubator model and use it to develop and ‘hatch’ novel community interventions with the potential to promote health, in sectors where health disparities are most apparent. The approach can be considered a form of CBPR in its aim to develop community partnerships and foster innovative interventions to improve health [15]. However Project Raphael differed in its scope, in that it worked broadly with a number of organisations, who had generated their own ideas. The organisations had full ownership, while the medical school took more of a supportive role, and the focus was less on research, although a rigorous evaluation process was developed for each project.

There are a number of levels by which one can judge whether this novel incubator was a success. Of the 13 participating projects all but one was implemented. Three subsequently showed real proof of concept, demonstrated by community partners’ attaining ongoing competitive funding from national and international sources. Their innovative low-cost ideas targeting disadvantaged populations did not only help secure funding, but were the main facilitator in sustaining these projects over time.

Unlike technological incubators, a social incubator is not likely to create a platform for academia and business, nor promote the emergence of new technology-based growth firms [27], however it was clear that ‘value added ties’ [20, 21] were promoted. The Medical Faculty mentored the projects and contributed knowledge on what was happening in the field at large, while gaining an understanding of current local efforts at the grassroots level. Community partners gained knowledge, skills and credibility and the opportunity to develop, launch and implement innovative projects. These ties were not limited to the three projects that successfully hatched; all of the projects benefited and were sustained to different extents in their own settings.

The concept of a social incubator should be viewed in the context of CBPR. Wallerstein and Duran (2010) [28], described the challenges of CBPR partnerships, which included common barriers of lack of trust, difficulty in changing traditional perceptions, uni-directional flow of knowledge from academia to the community and difficulty in achieving sustainability. Our experience with Raphael indicated that trust was readily achieved and knowledge exchange between the partners was certainly bi-directional. Sustainability inevitably proved to be a challenge but, as discussed, we were well satisfied by the degree of sustainability that was attained.

The overarching aim of Raphael was to contribute to health by providing solutions that had the potential to reduce disparities across the Region. It was gratifying that the first step of partnering with appropriate organisations was reached; incubator partnerships were established with non-profit organisations working with disadvantaged and harder to reach local populations, particularly Arab and ultra-orthodox (Haredi) communities, the disabled and those suffering from mental illness. As hoped, the application process with 90 responses allowed preliminary mapping of the health concerns that preoccupy community organisations. It seemed more than appropriate that so many projects focused on lifestyle and emotional wellbeing, given the widespread problems of non-communicable diseases and mental illness, which differentially afflict the poor and disadvantaged.

Two of the incubator’s aims were less adequately fulfilled. The attempt to foster a network of health promoting organisations with the medical school at the hub failed, principally because distances mitigated against meeting, even periodically, to share ideas and progress. Our assumption that partnership working with an academic team would engender capacity building in the partner organisations was also not realised. While most of the projects involved specific training relating to the project aims within the organisations, there was less evidence that staff were using more generic skills to develop their organisations.

On a final note, there was an additional benefit that is worthy of mention. The medical school was less than a year old when Project Raphael was conceived. With a strong focus on medical education and development of laboratory based medical science, it was already in danger of becoming a remote ivory tower on the hill. There is no doubt that Project Raphael averted this and successfully opened the medical school’s doors to the local community and engendered meaningful connections which have borne fruit.

## Conclusions

Project Raphael was devised as a ‘social incubator’ adapting the concept of technological incubators [21] as its model. It aimed to tackle health disparities in a disadvantaged region by adopting a ‘bottom up’ approach [2] and nurturing novel ideas engendered by community organisations. Judging the experiment in the light of technological incubators it was a success, in the short term at least. Using Rothschild and Darr’s criteria for technological incubators [21], Project Raphael led to a combination of practices and professional norms, promoted ‘value added ties’ in which the medical school took a mentoring role, offered access to academic expertise and knowledge on what was happening in the field, and led to a gain in knowledge, skills and credibility within the organisations.

**Table:** 

LESSONS LEARNT • The concept of a ‘social incubator’ is a feasible and novel way to create productive academia-community partnerships while demanding minimal resources. • The process led community partners to develop stronger connections with key stakeholders such as municipalities, other NGOs, and local government offices. • The diversity of the population, organisations and locations enriched the process and mutual knowledge of local needs. • A strength of the academic team was its multidisciplinary nature, knowledge of the area and culture, and local languages. • The time frame required is greater than 12 months for adequate design and implement of projects. • The partnership needs to address scalability as well as sustainability. • The process embedded the Faculty in the community and created a platform for its social accountability.	

## Data Availability

Not applicable.

## Abbreviations

NGO: Non-governmental organization
CBPR: Community Based Participatory Research
CIROP: Community Impact of Research Oriented Partnerships
